# Structure and Optical Properties of Titania-PDMS Hybrid Nanocomposites Prepared by In Situ Non-Aqueous Synthesis

**DOI:** 10.3390/nano7120460

**Published:** 2017-12-20

**Authors:** Antoine R. M. Dalod, Ola G. Grendal, Anders B. Blichfeld, Vedran Furtula, Javier Pérez, Lars Henriksen, Tor Grande, Mari-Ann Einarsrud

**Affiliations:** 1 Department of Materials Science and Engineering, NTNU, Norwegian University of Science and Technology, 7491 Trondheim, Norway; antoine.r.m.dalod@ntnu.no (A.R.M.D.); ola.g.grendal@ntnu.no (O.G.G.); anders.b.blichfeld@ntnu.no (A.B.B.); tor.grande@ntnu.no (T.G.); 2 Department of Physics, NTNU, Norwegian University of Science and Technology, 7491 Trondheim, Norway; vedran.furtula@ntnu.no; 3 SWING Beamline at Synchrotron SOLEIL, L’Orme des Merisiers Saint-Aubin BP 48, 91192 Gif-sur-Yvette, France; javier.perez@synchrotron-soleil.fr; 4 poLight AS, Kongeveien 77, 3188 Horten, Norway; lars.henriksen@polight.com

**Keywords:** hybrid material, organic-inorganic, nanocomposite, titania, TiO_2_, polydimethylsiloxane (PDMS), non-aqueous, sol-gel, refractive index

## Abstract

Organic-inorganic hybrid materials are attractive due to the combination of properties from the two distinct types of materials. In this work, transparent titania-polydimethylsiloxane hybrid materials with up to 15.5 vol. % TiO_2_ content were prepared by an in situ non-aqueous method using titanium (IV) isopropoxide and hydroxy-terminated polydimethylsiloxane as precursors. Spectroscopy (Fourier transform infrared, Raman, Ultraviolet-visible, ellipsometry) and small-angle X-ray scattering analysis allowed to describe in detail the structure and the optical properties of the nanocomposites. Titanium alkoxide was successfully used as a cross-linker and titania-like nanodomains with an average size of approximately 4 nm were shown to form during the process. The resulting hybrid nanocomposites exhibit high transparency and tunable refractive index from 1.42 up to 1.56, depending on the titania content.

## 1. Introduction

The progress in organic-inorganic hybrid materials with advanced and multifunctional properties [1,2,3] has led to the development of new materials for various optical applications [4,5,6,7,8,9,10,11]. The versatility of the sol-gel chemistry offers wide possibilities for synthesis of organic-inorganic materials with desired contents and nanostructures [12,13]. Hybrid materials based on polydimethylsiloxane (PDMS) have been widely investigated to tune for example the mechanical properties [14,15], the refractive index [16,17] and the hydrophobicity [18,19]. A large family of metal-polydimethylsiloxane nanocomposites (M-PDMS) using metal alkoxides M(OR)*_z_* (M = Si, Ti, Zr, Ge, Nb, V, Al, Ta) as precursors have been prepared via various sol-gel approaches [14,15,20,21,22,23,24,25,26,27]. One approach is based on in situ co-polymerization of alkoxysilane such as tetraethoxysilane (TEOS) and/or dimethyldiethoxysilane (DMDES) and metal alkoxide precursors. Using metal alkoxides with a high degree of unsaturation, e.g., Ti(IV) and Zr(IV), phase separation into hydrophobic PDMS regions and hydrophilic metal-rich domains has been observed depending on the M/PDMS ratio [22,25,26]. An alternative strategy is to use “pre-polymerized” precursors such as hydroxy-terminated PDMS (PDMS-OH), which thus reduces the complexity of having simultaneous hydrolysis and condensation mechanisms with important differences of reaction kinetics. In several works, ethyl acetoacetate has been used as metal alkoxide modifier to reduce the hydrolysis reaction rate [14,17,20,28]. By using this sol-gel method, Yamada et al. [14] prepared M-PDMS (M = Al, Ti, Ta) and investigated the effect of the thermal treatment, the PDMS-OH precursor molecular mass and the nature of the metal alkoxide on the mechanical properties. Higher PDMS molecular mass and higher valence of the metal cation (Al^3+^, Ti^4+^, Ta^5+^) resulted in higher storage modulus. Almeida et al. [29] proposed another synthesis route in which no ethyl acetoacetate was used, leading to a drastic reduction of the colorization of the M-PDMS (M = Ti, Zr) materials observed using ethyl acetoacetate. However, the synthesis required a high pH of 10–13 and two weeks of reaction time. Lu and Mullins [16] proposed a preparation method in which, contrary to previous methods, no inhibitor nor water was used, greatly simplifying the synthesis of Ti-PMDS materials. The strategy resides in the use of isopropanol as solvent, being also the product of the hydrolysis reaction of titanium(IV) isopropoxide (TIP) and shifting the reaction towards stabilization of the TIP precursor. The cross-linking reaction is then induced by evaporation of the solvent followed by a curing step. By this method, Ti-PDMS films were produced with refractive index (at 600 nm) from 1.45 to 1.67, using TIP/PDMS molar ratios from 5:1 to 20:1, respectively. However, the materials were solely analysed by infrared spectroscopy and ellipsometry and the nanostructure of the materials was not investigated in detail.

In this work, we extended the sol-gel procedure described by Lu and Mullins [16] by using different PDMS-OH precursors and various TIP/PDMS-OH molar ratios (i.e., 25 cSt, 50 cSt and 750 cSt and 2:1, 5:1, 10:1, 15:1 and 20:1, respectively) to prepare mould-casted and spin-coated Ti-PDMS hybrid materials. The samples are labelled Ti-PDMS-*X*-*Y*:1, where *X* is the viscosity of the PDMS-OH precursor (in cSt) and *Y* is the molar ratio of TIP/PDMS-OH. The theoretical content of TiO_2_ was calculated to range between 0.2 and 15.5 vol. %, assuming full reaction of the titanium precursor to anatase TiO_2_ (see Section 3). The optical properties of the nanocomposites such as transparency and refractive index were determined. Moreover, the structure of the nanocomposites was thoroughly investigated by spectroscopy (Fourier transform infrared, Raman, Ultraviolet-visible, ellipsometry) and small-angle X-ray scattering analysis and discussed in relation to the total titania content. Finally, potential applications and further possible developments of these materials and the synthesis method, respectively, are discussed.

## 2. Results and Discussion

### 2.1. Structure

**Figure 1:**
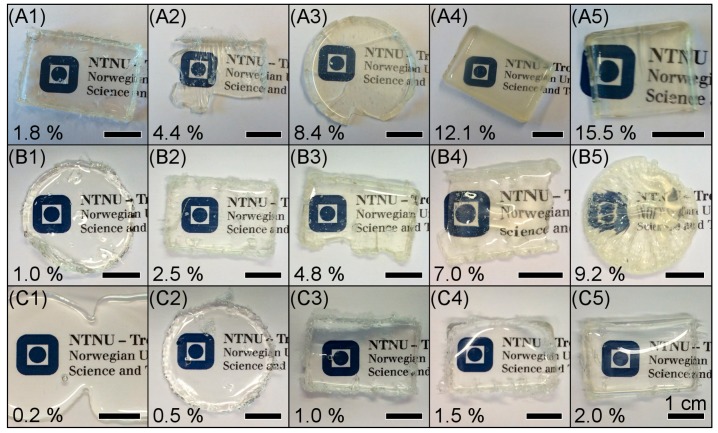
Photographs of mould-casted Ti-PDMS hybrid films prepared using PDMS-OH with viscosities of (**A**) 25 cSt, (**B**) 65 cSt and (**C**) 750 cSt; and TIP/PDMS-OH molar ratios of (**1**) 2:1, (**2**) 5:1, (**3**) 10:1, (**4**) 15:1 and (**5**) 20:1. The circular and rectangle shaped samples of Ti-PDMS-750-20:1 (**C1**) had merged. The scale bars are 1 cm for all images and the percentages indicate the volume content of TiO_2_.

Figure 1 shows photographs of the films prepared by mould casting. The thickness of the films varied between 1 and 2 mm. All the films are transparent with a yellow tint at high TiO_2_ content and increases as a function of increasing volume fraction of TiO_2_. Higher volume fraction of titanium precursor led to more brittle materials, which caused partial damage of the films during demoulding. The rectangular and circular films of Ti-PDMS-750-20:1 (Figure 1C1) had merged, however, these samples were substantially more viscous than the PDMS-OH precursor and the film could still be held with a pair of tweezers, indicating that a partial cross-linking had occurred. The PDMS-OH precursors are most likely not monodisperse and the amount of TIP was in consequence not sufficient to provide the formation of a continuous network. The merging of the samples by viscous flow was observed after a few days.

**Figure 2:**
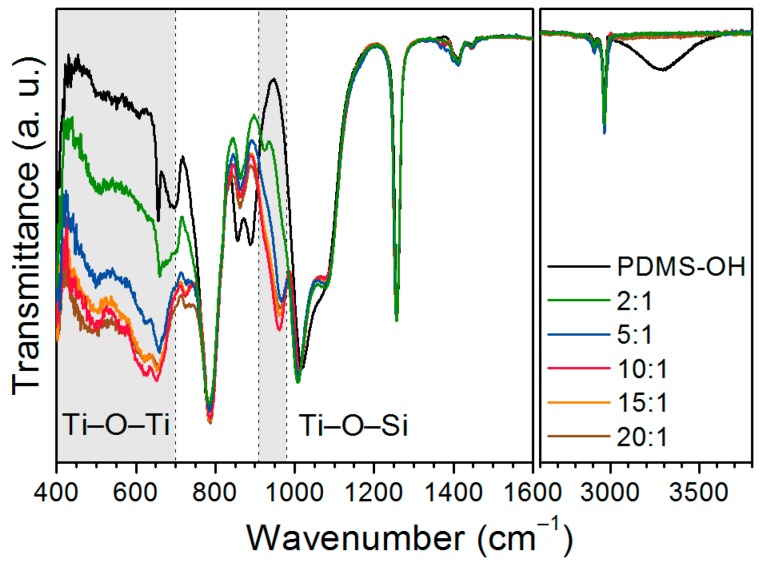
FTIR spectra of Ti-PDMS hybrid materials prepared using PDMS-OH precursors with viscosities of 25 cSt for different TIP/PDMS-OH molar ratios. The spectra are normalized to the Si–CH_3_ band at 1260 cm^−1^.

**Figure 3:**
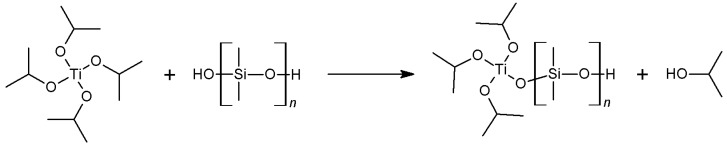
Scheme of the initial step involved in the reaction of TIP with PDMS-OH. The remaining isopropoxy groups can further react with PDMS-OH or with TIP to form titania-like domains.

Fourier transform infrared (FTIR) spectra of the 25 cSt PDMS-OH precursor and the Ti-PDMS series based on this precursor are shown in Figure 2 and the assignments of the spectra are provided in Table 1. The characteristic absorption bands of PDMS are observed for all materials. In addition, the broad band centred at 3300 cm^−1^ (terminal –OH groups) present in the PDMS-OH precursor is not visible for the hybrid films, confirming full reaction of the –OH groups of the PDMS-OH precursor as well as complete evaporation of the solvent. The absorption in the 910–990 cm^−1^ region and below 700 cm^−1^ increased with increasing TIP/PDMS-OH ratio. These bands are attributed to Ti–O–Si and Ti–O–Ti vibrations, respectively, showing that cross-linking of the PDMS chains with the titanium alkoxide had occurred (Figure 3) in addition to the formation of titania-like domains. The series using 50 cSt and 750 cSt PDMS-OH precursors exhibited similar behaviour but less pronounced as the amounts of incorporated titanium alkoxides and terminal –OH groups are lower (Figure S1). In the synthesis described here, TIP was used in large excess compared to the number of available terminal –OH groups of the PDMS precursor and the formation of titania-like domains was desirable. Furthermore, in the case of using water/ethanol during the synthesis, it has previously been shown that titanium alkoxide could also catalyse the formation of longer PDMS segments, resulting in phase separation of PDMS-rich domains and titania-like domains [25,26]. The band in the 910–990 cm^−1^ region was assigned to Ti–O–Si (Figure 2) and the materials with the lowest TIP content exhibit a maximum at about 925 cm^−1^ which shift towards higher wavenumber (about 960 cm^−1^) with increasing TiO_2_ content. In titanosiloxane oligomers or Ti-PDMS hybrids, the Ti–O–Si band is often observed at around 925 cm^−1^ [17,30,31,32]. However, the position of a band is influenced by the rigidity of the bond and by the environment, as the Ti–O–Si band was measured and predicted at about 960 cm^−1^ for titanium in a zeolite-like structure [33]. The inorganic domains are amorphous (Figure S2) and titanium could thus have various coordination numbers in the form of titania-like species covalently linked to PDMS chains. A combination of various coordination numbers of titanium and increased rigidity could explain the shift of the Ti–O–Si absorption at higher TiO_2_ contents.

**Figure 4:**
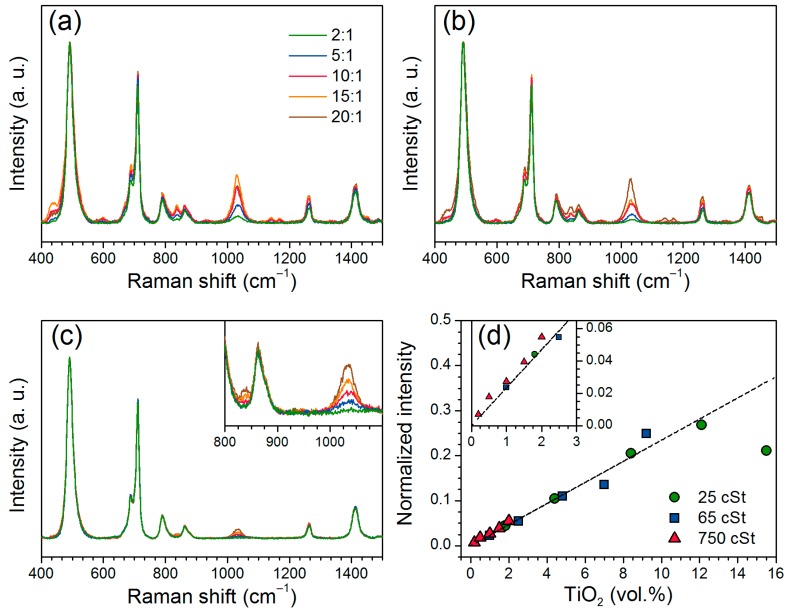
Raman spectra of Ti-PDMS hybrid materials prepared using PDMS-OH precursors with viscosities of (**a**) 25 cSt, (**b**) 50 cSt and (**c**) 750 cSt for different TIP/PDMS-OH molar ratios. The spectra are normalized to the Si–O–Si band at 495 cm^−1^. (**d**) Normalized intensity of the band at 1030 cm^−1^ assigned to asymmetric stretching of Ti–O–Si bonds as a function of the TiO_2_ vol. %.

Additional Raman analysis was performed because the Si–O–Si doublet in the 1000–1100 cm^−1^ is non-active in Raman spectroscopy [23], which reduces the number of overlapping bands. Raman spectra of the Ti-PDMS hybrids are displayed in Figure 4a–c and corresponding assignments are included in Table 1. The characteristic PDMS absorption bands are observed in the Raman spectra. Two additional bands appeared at 835 and 1030 cm^−1^, were assigned to symmetric and antisymmetric Ti–O–Si stretching, respectively, by comparison to similar work on V-PDMS materials [23] and numerical simulations of titania-silica systems [33]. When normalizing the Raman spectra to the Si–O–Si band at 495 cm^−1^, a linear relation between the relative intensity of the Ti–O–Si band at 1030 cm^−1^ and the volume content of TiO_2_ is observed, even at very low TiO_2_ contents (Figure 4d). Thus, Raman spectroscopy is an efficient method to quantify titania-like species in the in situ prepared titania-polysiloxane materials. At high titania content (Figure 4a,b), additional bands attributed to additional phases such as amorphous titania at 600 cm^−1^ [41] or additional interactions such as the band at 440 cm^−1^, previously assigned to Si–O–Si in a more rigid environment [22] are observed.

**Figure 5:**
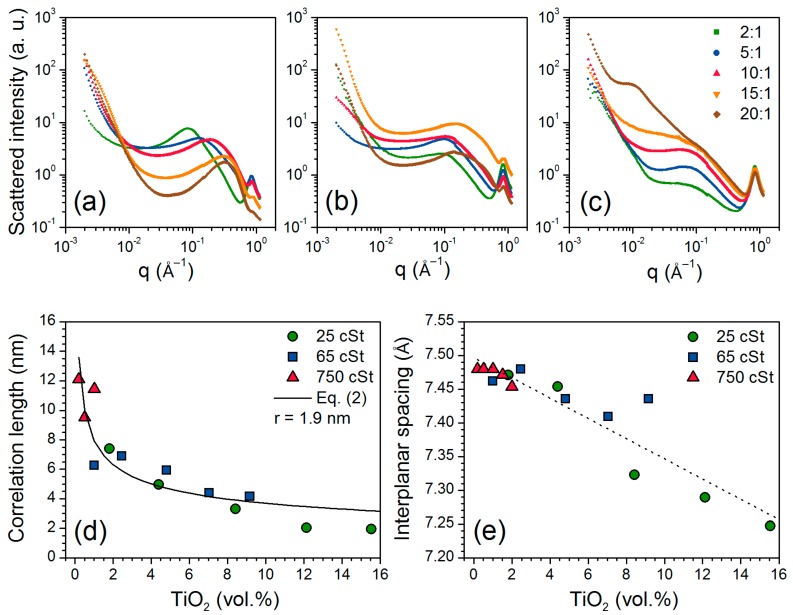
SAXS profiles (*λ* = 1.0332 Å) of mould-casted Ti-PDMS hybrid films prepared using PDMS-OH with viscosities of (**a**) 25 cSt, (**b**) 50 cSt and (**c**) 750 cSt for different TIP/PDMS-OH molar ratios. (**d**) Correlation length of titania-like inorganic amorphous nanodomains with fitting to Equation (2) and (**e**) interplanar spacing of the PDMS amorphous halo as a function of the TiO_2_ vol. % with linear regression.

Small-angle X-ray scattering (SAXS) profiles (Figure 5a–c) display a broad maximum assigned to the correlation length between inorganic amorphous titania-like nanodomains, as previously observed for in situ synthesized nanocomposites [22,24,42,43]. The correlation length (*ζ*) was calculated using [22,42,43]:

(1) $$\zeta =\frac{2\pi }{q_{\max}}$$

where *q*_max_ is the scattering vector (*q*) at the maximum of the broad peak. Measured *q*_max_ and calculated *ζ* values are reported in Table 2. Figure 5d shows the evolution of *ζ* as a function of the volume content of TiO_2_. Assuming homogeneously dispersed spherical titania-like regions, an average radius of the nanodomains (*r*) can be estimated using [44]:

(2) $$\zeta =0.55396{\left(\frac{4\pi }{3\varphi }\right)}^{1/3}r$$

where *φ* is the volume fraction of titania-like nanodomains. The correlation lengths were fitted to Equation (2) and the calculated average size of the titania-like nanodomains is 3.8 nm (*r* = 1.9 nm, Figure 5d). For these calculations, samples Ti-PDMS-750-15:1 and Ti-PDMS-750-20:1 were not included as the broad maximum (Figure 5c) is not clearly defined but a shoulder is visible at a similar position (0.1 Å^−1^) compared to the other samples in the series. In addition, for Ti-PDMS-750-20:1, another broad maximum centred at about 0.01 Å^−1^, can be assigned to larger scale inhomogeneities (*ζ* = 63 nm), indicating a hierarchical and/or disperse structure of the titania-like nanodomains. The calculated average nanodomain size is consistent with the high transparency of the films (Figure 1), even at loads as high as 15.5 vol. % TiO_2_, confirming homogeneous distribution of titania within the nanocomposites at larger scales. The increased absolute values of the slopes (*D*) from about 1.4 to 3.5 (Table 2) observed in the linear region at low *q*-range of the log-log SAXS profiles (left of the broad correlation peak, Figure 5a–c) suggest an increased fractal dimension of the surfaces of the nanocomposites and can be attributed to an increased roughness of the surfaces as the titania content increased [42,43,45]. At high *q*-range (*q* ≈ 0.84–0.87 Å^−1^), the SAXS profiles also display PDMS amorphous halos commonly observed in X-ray diffraction [43,46] (Figure S2). The position of the halo (*q*_halo_) and the equivalent interplanar spacing (*d*) are given in Table 2. The relative intensity of the halo decreases as the titania content increases in the three series (less visible for 750 cSt series). Figure 5e shows the measured interplanar spacing of the PDMS amorphous halo from the SAXS analysis decreasing as a function of the volume content of TiO_2_, which can be due to more densely packed polymeric chains caused by the increased number of surrounding inorganic amorphous nanodomains.

**Figure 6:**
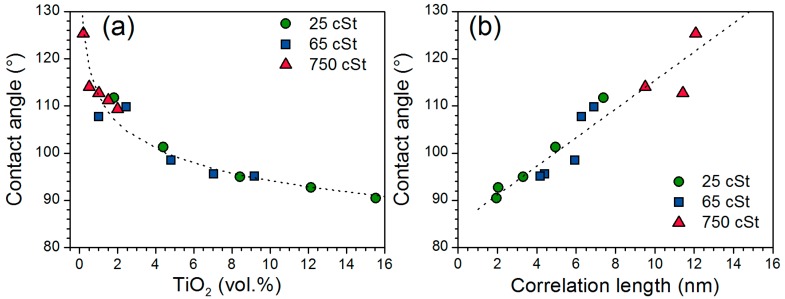
Contact angle of water (±1°) as a function of (**a**) the TiO_2_ vol. % of the hybrid materials with power regression and (**b**) the correlation length of the titania-like nanodomains measured by SAXS with linear regression.

The contact angle of water measured on the spin-coated films (Figure S3) are included in Table 2 and are displayed in Figure 6a as a function of the volume content of TiO_2_. The contact angle decreases from 126° to 91° as more titania was incorporated in the nanocomposites, showing an increased hydrophilicity of the hybrid materials due to the respective contributions of the hydrophobic PDMS and hydrophilic titania-like species. The ratio of surface area of PDMS and titania-like phase is directly related to the correlation length between the nanodomains, assuming a constant nanodomain size. A linear relationship is observed between the contact angle of water and the correlation length of the titania-like nanodomains, *ζ*, within the experimental uncertainty of both analysis (Figure 6b).

### 2.2. Optical Properties

**Figure 7:**
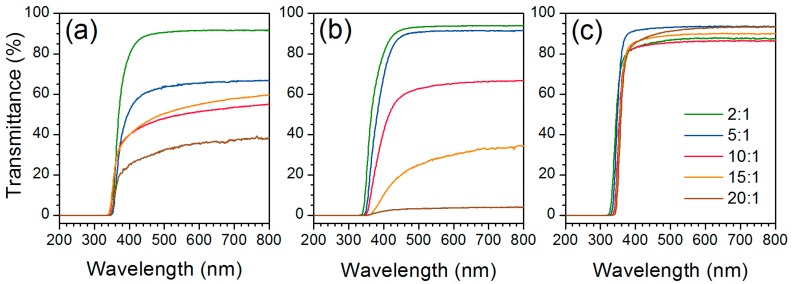
UV-vis spectra of mould-casted Ti-PDMS hybrid films (shown in Figure 1) prepared using PDMS-OH with viscosities of (**a**) 25 cSt, (**b**) 65 cSt and (**c**) 750 cSt for different TIP/PDMS-OH molar ratios.

Ultraviolet-visible (UV-vis) spectra measured in transmission mode of mould-casted films (Figure 1) are displayed in Figure 7, confirming the transparency in the visible region of the spectrum (400–800 nm), up to 94% at 600 nm (1–2 mm thickness). The transmittance is however significantly reduced with increasing titania content but the drastic drop of the transparency is also due to the light scattering induced by the relative roughness of the samples, especially in the case of Ti-PDMS-65-15:1 and Ti-PDMS-65-20:1 (Figure 1B4,B5, respectively) where the transparency at 600 nm were 30% and 4%, respectively. The transmittance is reduced to zero at 320–360 nm in the UV region. The onset where the transmittance is reduced to zero is red-shifted as the TiO_2_ content was increased. This red-shift results in a partial absorption in the blue region, thus the yellow colorization of the films with higher titania content.

**Figure 8:**
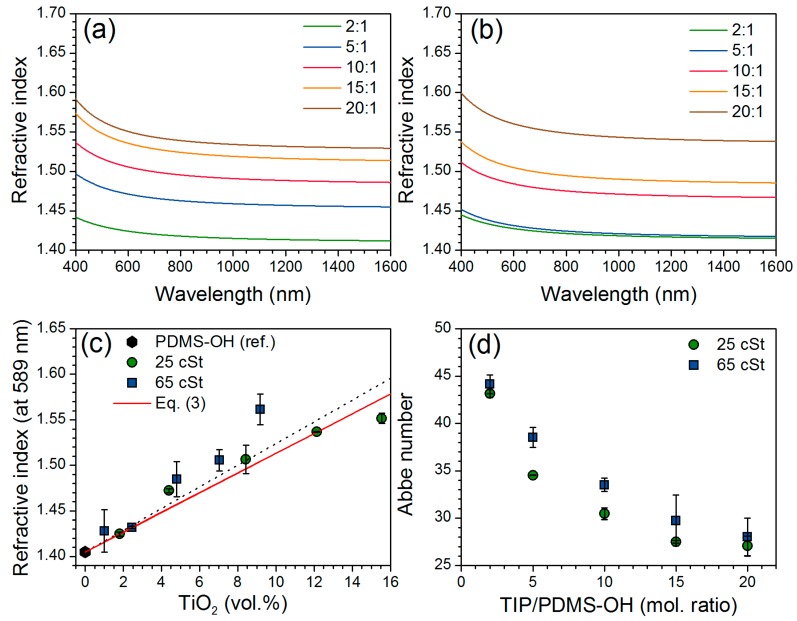
Average refractive index of spin-coated Ti-PDMS hybrid films prepared using PDMS-OH with viscosities of (**a**) 25 cSt, (**b**) 65 cSt for different TIP/PDMS-OH molar ratios, (**c**) refractive index at 589 nm as a function of the TiO_2_ vol. % of the hybrid materials with linear regression (reference point at 0 vol. % TiO_2_ is given by the PDMS-OH manufacturer) and comparison with Equation (3) and (**d**) Abbe number of spin-coated Ti-PDMS hybrid films as a function of the TIP/PDMS-OH molar ratio.

The refractive indices measured on the spin-coated films by ellipsometry are reported in Figure 8a,b for the 25 cSt and 50 cSt series, respectively. The refractive index decreased with increasing wavelength, in accordance with Cauchy’s law and both series exhibit an increase in the refractive index with increasing TIP/PDMS-OH molar ratio. At constant wavelength (*λ* = 589 nm, Figure 8c), the refractive index of the hybrid materials increases with the volume content of titania. Linear increase of the refractive index of polymer nanocomposites as a function of volume content of TiO_2_ have previously been observed [47,48,49,50,51]. A theoretical refractive index of the TiO_2_-PDMS nanocomposites *n*_comp_ can thus be estimated using [52,53]:

(3) $$n_{\mathrm{comp}}=\varphi_{{\mathrm{TiO}}_{2}}n_{{\mathrm{TiO}}_{2}}+\varphi_{\mathrm{PDMS}}n_{\mathrm{PDMS}}$$

where *φ* and *n* are the volume fraction and the refractive index, respectively and using 2.49 and 1.406 as refractive indices for anatase TiO_2_ [54] and PDMS-OH precursors (value given by the manufacturer), respectively. The refractive index of the hybrid materials was increased from 1.40 to 1.56 (11% increase) at 589 nm by incorporation of TiO_2_. Extrapolation to 100 vol. % TiO_2_ gives a refractive index of about 2.6, close to the theoretical value of TiO_2_. The refractive indices of the 25 cSt series were expected to be higher than the ones measured for the 65 cSt series, as they contain proportionally more titania. For this series, the drying and curing temperatures were lower, reducing the shrinkage of the network during solvent evaporation. In consequence, the density is probably lower resulting in a lower refractive index. However, the structure of the nanocomposites is also expected to vary because of the different nature of the PDMS-OH precursor and titanium alkoxide content used for each series. Due to the higher hydrophobicity of the 750 cSt PDMS-OH precursor, partial phase separation occurred during the spin-coating process, hence the quality of the films was not sufficient for accurate measurements by ellipsometry. However, this series contains the lowest content of TiO_2_ and the resulting nanocomposites would thus not be expected to exhibit a drastic increase in the refractive index. With higher titania content, the wavelength dependence of the refractive index becomes more significant and the Abbe number (Figure 8d) of both series decreased in a similar way, demonstrating an increased dispersion in the visible region of the light spectrum. Reduced Abbe number with increased titania content has previously been observed in in situ prepared nanocomposites [42,55].

In summary, thorough analysis of the Ti-PDMS materials demonstrated cross-linking of the PDMS chains by TIP via the formation of Ti–O–Si bonds and the formation of amorphous titania-like inorganic nanodomains, with an average size <4 nm, embedded in the PDMS network. Due to the amorphous nature of the titania-like nanodomains which drastically reduces the photocatalytic properties of titania [56,57], the stability of the nanocomposites under UV illumination is expected to be similar to that of pure PDMS. The properties of the nanocomposites, e.g., the refractive index, can be rationalized by the hypothesis that the titanium precursor was fully converted to anatase TiO_2_. This synthesis method has the advantage of requiring no inhibitor neither catalyst, by the combined use of anhydrous isopropanol and pre-hydrolysed polysiloxane precursor, respectively. The synthesis conditions, such as the composition and the temperatures of drying and curing were demonstrated to have a strong influence on the final morphology of the materials and the optimization of the process conditions is key in order to prepare homogeneous and transparent materials [10,14,24]. The refractive index of the nanocomposites was increased from 1.40 up to 1.56 and could be further increased by including high refractive index groups to the polymer precursor such as phenyl groups [6,58,59]. The synthesis method can be further extended by using different polymer precursors exhibiting terminal or branched –OH groups and/or other type of metal alkoxides.

## 3. Materials and Methods 

### 3.1. Synthesis

The polymeric precursors were hydroxy-terminated PDMS (PDMS-OH) with viscosities of 25, 65 and 750 cSt (Sigma-Aldrich, St. Louis, MO, USA), corresponding to average molecular masses of 2100, 4000 and 20,000 g mol^−1^, respectively [60]. For each PDMS-OH precursor, hybrid materials were prepared with titanium(IV) isopropoxide (TIP, ≥97%, Sigma-Aldrich, St. Louis, MO, USA) using TIP/PDMS-OH molar ratios of 2:1, 5:1, 10:1, 15:1 and 20:1 for which the calculated volume content of TiO_2_ was ranging from 0.2 to 15.5 vol. % (Table 3), assuming full reaction of the TIP precursor to anatase, the expected polymorph of TiO_2_ at nanoscale [61]. Thermogravimetric analysis did not allow for accurate measurement of the weight ratios between organic and inorganic components of the Ti-PDMS hybrids, as even after heating up to 1200 °C in air, black and glassy materials resulted, demonstrating residual carbon in the TiO_2_-SiO_2_ materials (Figure S4).

The synthesis route was optimized for all of the series as follow. In a first step, PDMS-OH (3 mL) was mixed with 8 mL anhydrous propan-2-ol (AIP, 99.5%, Sigma-Aldrich, St. Louis, MO, USA) in a closed glass container. The solution was sonicated for 5 min at room temperature and subsequently stirred and heated at 70 °C. In another closed glass container, the necessary volume of TIP was added to AIP (4 mL) and sonicated for 5 min at room temperature. The titanium precursors were then mixed with the PDMS-OH precursors and the obtained sols were vigorously stirred for 30 min at 70 °C while keeping the containers closed in order to avoid solvent evaporation. The sols were either spin-coated on single crystal Si wafers (1 × 1 cm) for 1 min at 3000 rpm, including 15 s of acceleration and 15 s of deceleration or mould-casted in rectangular and circular polytetrafluoroethylene moulds. The films were dried at 50 °C for 24 h and cured at 100 °C for 24 h on a hot plate. The 25 cSt series was dried at room temperature and cured at 50 °C due to bubble and crack formation at higher temperatures.

### 3.2. Characterization

Fourier transform infrared (FTIR) spectra were acquired on a Bruker (Billerica, MA, USA) Vertex 80v spectrophotometer equipped with Bruker Platinum ATR diamond system. A total of 64 scans were acquired for each sample at a resolution of 1 cm^−1^.

Raman spectra were recorded on a Renishaw (Wotton-under-Edge, UK) InVia Reflex spectrometer using a monochromatic diode laser (*λ* = 785 nm) as an exciting light source and a 1200 L mm^−1^ grating. The spectra were measured on the mould-casted films, from 400 to 1500 cm^−1^, with a resolution of 1 cm^−1^. A total of 20 scans were collected with an acquisition time of 1 s and averaged for every sample.

Small-angle X-ray scattering (SAXS) data were collected at the SWING beamline, SOLEIL synchrotron (Gif-sur-Yvette, France) [62]. The acquisitions were performed in transmission mode, at sample-to-detector distances of 6.072 and 0.997 m, for accessing *q*-ranges (*q* = 4π sin(*θ*)/*λ*, where 2*θ* is the scattering angle and *λ* is the X-ray wavelength) from 0.002 to 0.2 Å^−1^ and from 0.015 to 1.2 Å^−1^, respectively, using a monochromatic beam with a wavelength of 1.0332 Å (12.000 keV). For each sample, the acquisition time was optimized in order to obtain high signal without saturating the detector. Once desired acquisition time was selected, 10 backgrounds (air and, in some cases, Kapton (DuPont, Wilmington, DE, USA) films) were collected, masked, integrated and averaged. The same number of acquisitions and treatments were performed for the sample data. All the data treatments were performed at the beamline using FOXTROT [63]. Due to overlapping *q*-ranges, *q* = 0.15 Å^−1^ was selected as the merging point and the values below and above are from the data collected at sample-to-detector distances of 6.072 and 0.997 m, respectively.

Contact angle measurements (sessile drop test) were performed on the spin-coated films, using a demineralized water drop of 8 µL at room temperature with a Krüss (Hamburg, Germany) Drop Shape Analyser-DSA 100. The Young-Laplace fitting was used on every image and the reported values are the average of 11 images (±1°).

Ultraviolet-visible (UV-vis) spectra were acquired with a Thermo Fisher Scientific (Waltham, MA, USA) Evo220 spectrophotometer from 1000 to 200 nm with a step size of 1 nm and an integration time of 0.1 s. A background was collected at room temperature in air and the mould-casted hybrid films were measured without substrate.

Ellipsometry analysis were performed on a J.A. Woollam (Lincoln, NE, USA) RC2 vertical ellipsometer. Calibration was achieved using a single crystal silicon wafer with a 25 nm SiO_2_ layer, at a position of 65° (source-sample angle, corresponding to 130° source-detector angle). Spin-coated films were analysed with a polychromatic light (210 to 1690 nm) using focusing probes. Three points per sample were recorded and for each point, measurements were done at five angles (50, 55, 60, 65 and 70° source-sample angles, corresponding to 100, 110, 120, 130 and 140° source-detector angles, respectively) with an analysis time of 20 s per position. The refinement of the data was performed using CompleteEASE (J.A. Woollam, version 5.13). The Cauchy model was used from 400 to 1600 nm on the hybrid film layer and the substrate was modelled with a 2 nm SiO_2_ layer on metallic silicon.

## 4. Conclusions 

Transparent Ti-PDMS hybrid materials with various titania content were prepared via an in situ non-aqueous method. Complementary characterization techniques allowed to describe the structure of the materials which formed a titania-PDMS network, in addition to embedded amorphous titania-like nanodomains (<4 nm). The average correlation length of the amorphous titania-like nanodomains demonstrated good correlation with the hydrophobicity of the hybrid materials. Furthermore, Raman spectroscopy was shown to be an efficient method to quantify the TiO_2_ content. Finally, the refractive index increased from 1.40 up to 1.56 with increasing titania content. The high refractive index and the transparency in the visible region make the Ti-PDMS materials developed in this work potential candidates for coatings and optical applications such as LED encapsulation [6,64] and waveguides [7,65].

## Figures and Tables

**Table 1:** Assignments of characteristic FTIR and Raman bands of PDMS-OH precursors and Ti-PDMS hybrid materials.

Assignment	Wavenumber (cm^−1^)		Sample	Reference
	FTIR	Raman		
Si–O–Si (stretch)	n/a	495	All	[22,23,34]
Ti–O–Ti	<700	n/a	Ti-PDMS	[35,36]
Si–CH_3_ (rock)	660–700	690	All	[23,34,37,38]
Si–C and CH_3_ (rock)	790	790	All	[22,23,34,37]
CH_3_ (rock)	860	860	All	[22,23,34,37,38,39]
Si–OH	890	−	PDMS-OH	[37,39]
Ti–O–Si	920–960	See text	Ti-PDMS	[22,30,31,35,40]
Si–O–Si (stretch)	1010 and 1080	n/a	All	[22,23,34,35,37,38,39]
Si–CH_3_	1260	1260	All	[22,35,37,38,39]
CH_3_ (bend)	1410 and 1440	1410	All	[22,23,34,35,37,38,39]
CH_3_ (stretch)	2900 and 2960	−	All	[22,23,34,35,37,38,39]
OH	3000–3600	−	PDMS-OH	[35,37,39]

**Table 2:** Structural properties of the Ti-PDMS hybrid materials obtained from SAXS and water contact angle analysis.

PDMS-OH	TIP/PDMS-OH (mol. Ratio)	*q*_halo_^a^ (Å^−1^)	*d* ^b^ (Å)	*q*_max_^c^ (Å^−1^)	*ζ* ^d^ (Å)	*D* ^e^	CA ^f^ (°)
25 cSt (*M* ≈ 2100)	2:1	0.841	7.47	0.085	73.92	1.4	112
	5:1	0.843	7.45	0.127	49.47	2.6	102
	10:1	0.858	7.32	0.190	33.07	2.8	95
	15:1	0.862	7.29	0.308	20.40	2.8	93
	20:1	0.867	7.25	0.321	19.57	2.8	91
65 cSt (*M* ≈ 4000)	2:1	0.840	7.48	0.091	69.05	1.9	110
	5:1	0.842	7.46	0.100	62.83	1.2	108
	10:1	0.845	7.44	0.106	59.28	1.4	99
	15:1	0.848	7.41	0.143	43.94	3.5	96
	20:1	0.845	7.44	0.151	41.61	3.0	96
750 cSt (*M* ≈ 20000)	2:1	0.840	7.48	0.052	120.83	2.0	126
	5:1	0.840	7.48	0.066	95.20	2.0	114
	10:1	0.840	7.48	0.055	114.24	2.7	113
	15:1	0.841	7.47	–	–	2.1	112
	20:1	0.843	7.45	0.010 ^g^	628.32 ^g^	2.7	110

^a^ Position of the PDMS amorphous halo from SAXS analysis. ^b^ Interplanar distance of the PDMS amorphous halo from SAXS analysis. ^c^ Position of the maximum of the correlation peak from SAXS analysis. ^d^ Correlation length of the titania-like nanodomains from SAXS analysis. ^e^ Slope (±0.1) at low *q*-range (<0.02 Å^−1^) from SAXS analysis. ^f^ Contact angle of water (±1°) measured on spin-coated films. ^g^ Assigned to a higher hierarchical degree (see text).

**Table 3:** Calculated weight and volume contents of TiO_2_ and Ti/Si atomic ratios of the Ti-PDMS hybrids, assuming full reaction of TIP to anatase.

PDMS-OH	TIP/PDMS-OH (mol. Ratio)	TiO_2_ (wt. %)	TiO_2_ (vol. %)	Ti/Si (mol. Ratio)
25 cSt (*M* ≈ 2100)	2:1	7.0	1.8	0.07
	5:1	15.9	4.4	0.18
	10:1	27.4	8.4	0.36
	15:1	36.1	12.1	0.54
	20:1	43.0	15.5	0.71
65 cSt (*M* ≈ 4000)	2:1	3.9	1.0	0.04
	5:1	9.2	2.5	0.09
	10:1	16.8	4.8	0.19
	15:1	23.3	7.0	0.28
	20:1	28.8	9.2	0.38
750 cSt (*M* ≈ 20000)	2:1	0.8	0.2	0.01
	5:1	2.0	0.5	0.02
	10:1	3.9	1.0	0.04
	15:1	5.8	1.5	0.06
	20:1	7.6	2.0	0.08

## Supplementary Materials

The following are available online at www.mdpi.com/2079-4991/7/12/460/s1, Figure S1: Infrared spectra, Figure S2: X-ray diffractograms, Figure S3: contact angle photographs, Figure S4: thermogravimetric analysis.
