# Supplementary material for: Structure and Optical Properties of Titania-PDMS Hybrid Nanocomposites Prepared by In Situ Non-Aqueous Synthesis
Source: Nanomaterials (Basel). 2017 Dec 20;7(12):460. doi: 10.3390/nano7120460 (PMC5746949; doi:10.3390/nano7120460)
Supplement: Supplementary file 1 [file nanomaterials-07-00460-s001.pdf]

# Supplementary Materials

# for

# Structure and Optical Properties of Titania-PDMS Hybrid Nanocomposites Prepared by *In Situ* Non-Aqueous Synthesis

**Antoine R. M. Dalod [1], Ola G. Grendal [1], Anders B. Blichfeld [1], Vedran Furtula [2], Javier Pérez [3], Lars Henriksen [4], Tor Grande [1] and Mari-Ann Einarsrud [1,*]**

[1] Department of Materials Science and Engineering, NTNU, Norwegian University of Science and Technology, 7491 Trondheim, Norway; antoine.r.m.dalod@ntnu.no (A.R.M.D.), ola.g.grendal@ntnu.no (O.G.G.), anders.b.blichfeld@ntnu.no (A.B.B.), tor.grande@ntnu.no (T.G.).

[2] Department of Physics, NTNU, Norwegian University of Science and Technology, 7491 Trondheim, Norway; vedran.furtula@ntnu.no (V.F.).

[3] SWING beamline at synchrotron SOLEIL, L'Orme des Merisiers Saint-Aubin BP 48, 91192 Gif-sur-Yvette, France; javier.perez@synchrotron-soleil.fr (J.P.).

[4] poLight AS, Kongeveien 77, 3188 Horten, Norway; lars.henriksen@polight.com (L.H.).

* Correspondence: mari-ann.einarsrud@ntnu.no; Tel.: +47-735-94-002 (M.-A.E.).

## 1. Fourier Transform Infrared Spectroscopy

Similar to the 25 cSt series (Figure 2 of the main text and Figure S1a), the 50 and 750 cSt series (Figure S1b-c) also exhibit the characteristic absorption bands of PDMS, as well as additional absorptions in the 910-990 $cm^{-1}$ region and below 700 $cm^{-1}$ which were assigned to Ti–O–Si and Ti–O–Ti vibrations, respectively, confirming the cross-linking of the PDMS chains by TIP and the formation of titania-like nanodomains. For the three series, the intensity of the Ti–O–Si and Ti–O–Ti bands increased as a function of increasing TIP/PDMS-OH ratio.

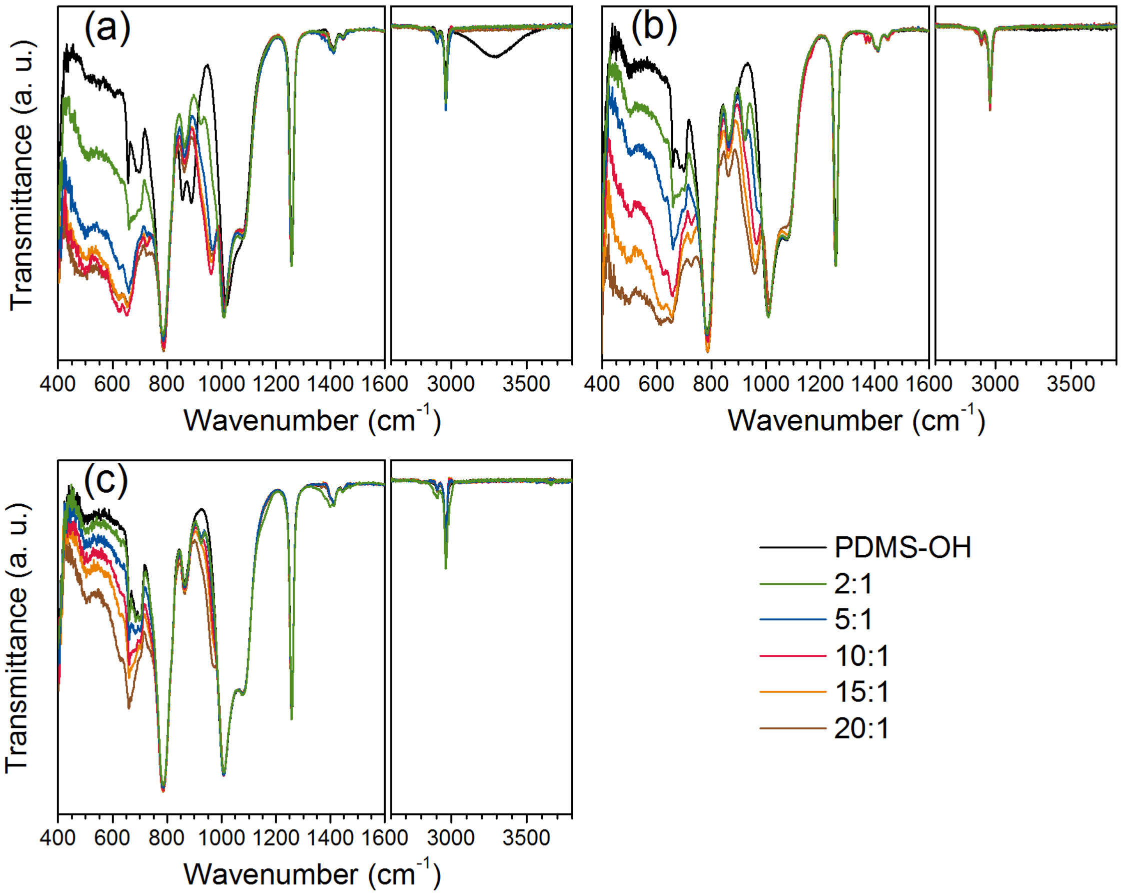


**Figure S1.** Infrared spectra of PDMS-OH precursors and Ti-PDMS hybrid materials prepared using PDMS-OH with viscosities of (**a**) 25 cSt, (**b**) 65 cSt, and (**c**) 750 cSt for different TIP/PDMS-OH molar ratios. The spectra are normalized to the Si–$CH_3$ band at 1260 $cm^{-1}$.

## 2. X-Ray Diffraction

The X-ray diffractograms (Figure S2) were recorded on a Bruker (Billerica, Massachusetts, United States) D8 Advance Da-Vinci equipped with LynxEye detector, with a step size of 0.05° and an integration time of 5.1 s. The intensity of the broad peaks centered at 12 and 21° decreased with increasing amount of TIP and are assigned to PDMS. No additional crystalline phase was observed with increasing TIP content, confirming the amorphous nature of the titania-like nanodomains.

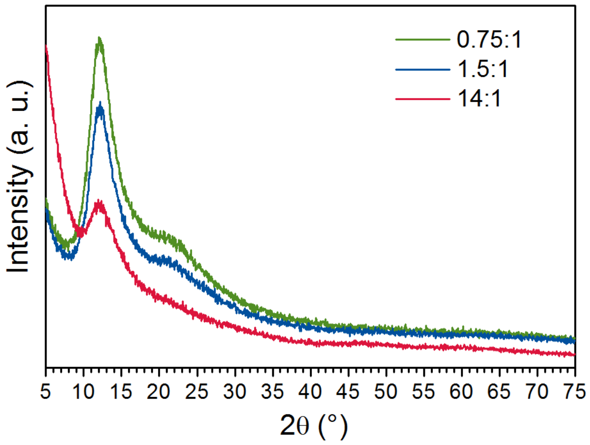


**Figure S2.** X-ray diffractograms ($\lambda_{Cu\text{-}K\alpha}$ = 1.5406 Å) of Ti-PDMS hybrid materials prepared using PDMS-OH with viscosities of 65 cSt and TIP/PDMS-OH molar ratios of 0.75:1, 1.5:1, and 14:1.

## 3. Water Contact Angle

Figure S3 shows selected photographs of water droplet contact angle for each Ti-PDMS composition, demonstrating the increased hydrophilicity of the materials as the TIP/PDMS-OH ratio was increased.

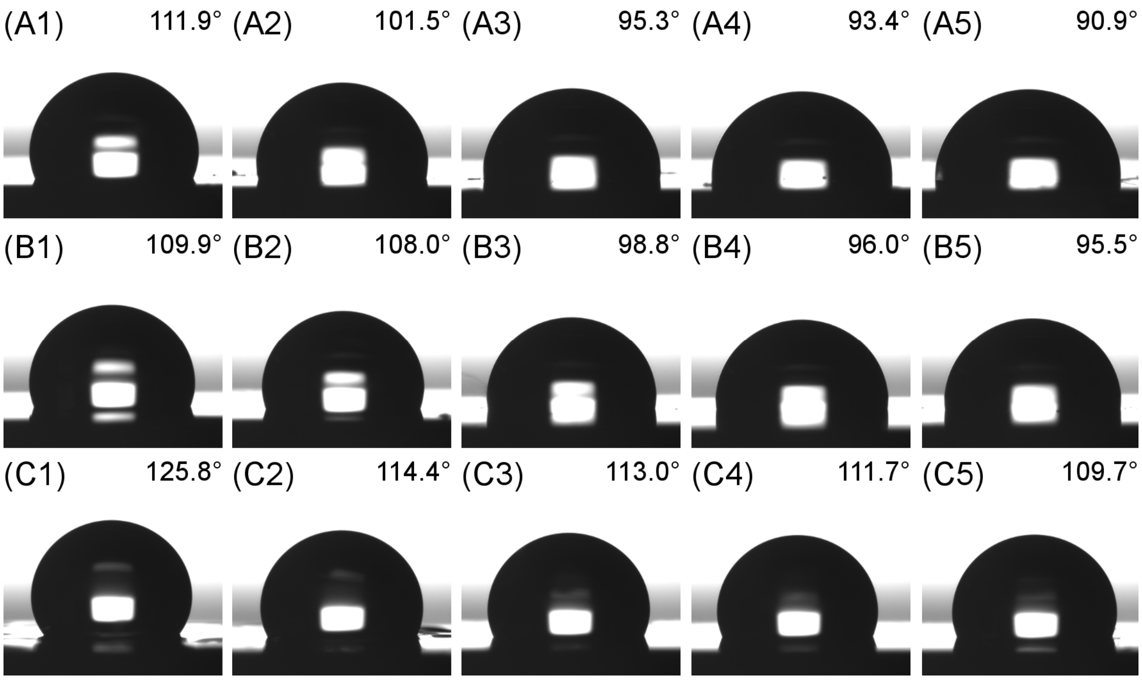


**Figure S3.** Example of photographs of water droplet contact angles measured on spin-coated Ti-PDMS hybrid films prepared using PDMS-OH with viscosities of (**A**) 25 cSt, (**B**) 65 cSt, and (**C**) 750 cSt; and TIP/PDMS-OH molar ratios of (**1**) 2:1, (**2**) 5:1, (**3**) 10:1, (**4**) 15:1, and (**5**) 20:1.

## 4. Thermogravimetric Analysis

Thermogravimetric analysis was acquired on a Netzsch (Selb, Germany) Jupiter STA 449 C using an alumina crucible in air. Analysis were performed on the Ti-PDMS hybrid materials (Figure S4a-b) in order to measure the organic/inorganic weight ratio for the 25 and 50 cSt series. The black color of the residual products (Figure S4c) demonstrates that some carbon remains trapped in the oxide network formed during the combustion which do not allow for accurate measurement of the organic/inorganic weight ratios. However, some trends may still be observed. For both series, the mass loss is reduced when the amount of incorporated TIP was increased, as the hybrids were made of more inorganic material. Similarly, the mass loss of the 25 cSt series is generally lower than for the 65 cSt series, and the difference between each molar ratio is greater in the 25 cSt series. This is consistent with the initial formulation of the films.

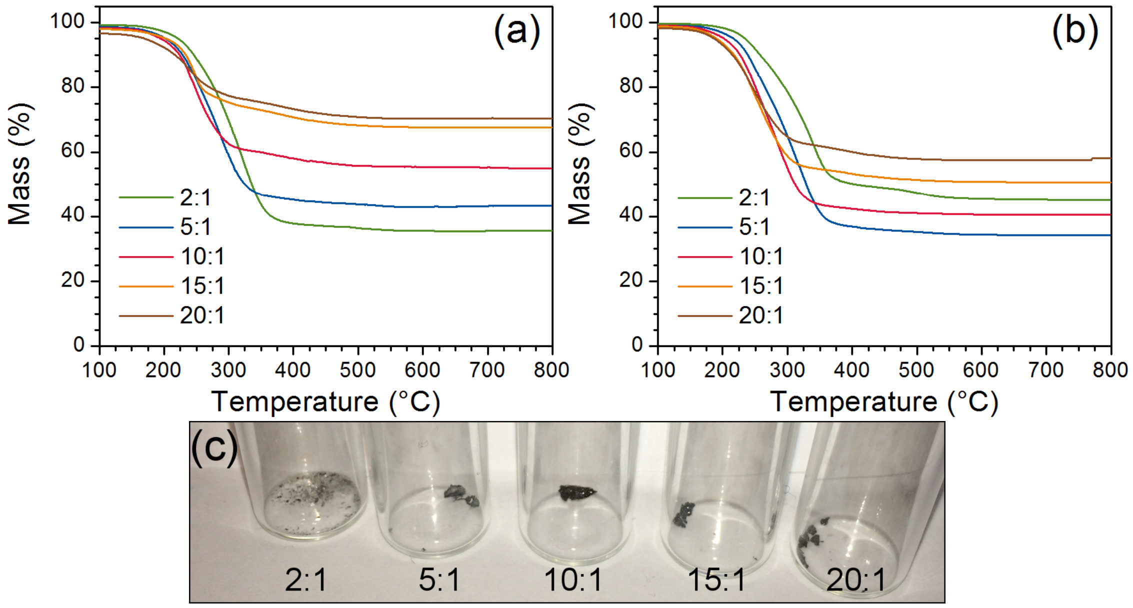


**Figure S4.** Thermogravimetric analysis on Ti-PDMS hybrid materials prepared using PDMS with viscosities of (**a**) 25 cSt and (**b**) 65 cSt for different TIP/PDMS-OH molar ratios; (**c**) picture of the residual products of the 65 cSt series after the analysis.
